# Supplementary material for: What do giant titanosaur dinosaurs and modern Australasian megapodes have in common?
Source: PeerJ. 2015 Oct 20;3:e1341. doi: 10.7717/peerj.1341 (PMC4662581; doi:10.7717/peerj.1341)
Supplement: Table S1 — Nesting sites selected for the current study correspond to the localities/areas that are highlighted in grey. AL, allochthonous; AU, autochthonous; PA, parautochthonous; X, known data; ?, imprecise information; -, unknown data. [file peerj-03-1341-s001.pdf]

Continent	Country	Region	Locality - Area*
**South America**	**Argentina**	Chubut	Huanimán
		Entre Ríos	Ita-i-cora
		La Rioja	Sanagasta
			Tama
		Neuquén	Auca Mahuevo
		Río Negro	Bajo de Santa Rosa- Salinas de Trapalcó
			Salitral Ojo de Agua, Salitral Moreno
			Yaminué
		Salta	
	**Perú**	Bagua	Fundo El Triunfo (Bagua)
		Laguna Umayo	Laguna Umayo
	**Uruguay**	Soriano	Palmitas
		Río Negro	Algorta
		Paysandú	Forestal Caja Bancaria Quarry
			El Quebracho
**Europe**	**Spain**	Southern Pyrenees	Ager syncline*
			Coll de Nargó syncline* / Pynies
			Tremp syncline*
			Vallcebre syncline*
	**France**	Aix Basin	Rennes-le-Château
			Albas
	**Romania**	Haţeg	Toteşti
**Asia**	**China**	Henan Province	Xixia
			Xiaguan
			Wulichuan
	**India**	Gujarat	Dholi Dungri
			Kehempur, Rahioli, Balasinor, Daulatpoira, Paori and Dholidhanti
		Madhya Pradesh	Bagh-Padalya-Borkui, Dholiya, Padiyal, Walpur-Kulwat, Jabalpur
		Maharashtra	Takli, Pisdura, Pavna
	**Mongolia**	Gobi	Algui Ulaan Tsav
	**South Korea**	Southern South Korea	Boseong
		Mid-west South Korea	Sihwa - Whaseong

Formation	Age - Stage	Taxon
Cerro Barcino	Aptian-Albian	Titanosauriformes?
Puerto Yaruá	Cretaceous	Titanosauria
Los Llanos	Hauterivian?-Cenomanian?	Titanosauria
Los Llanos	Campanian	Titanosauria
Anacleto	Campanian	Lithostrotia
Allen	Campanian - Maastrichtian	Titanosauria
Allen	Campanian - Maastrichtian	Titanosauria
Allen	Campanian - Maastrichtian	Titanosauria
Los Blanquitos	Coniacian? - Campanian	Titanosauria?
Bagua	Late Campanian - Maastrichtian	Titanosauria?
Umayo (now Muñani Fm.)	Late Cretaceous?	Titanosauria?
Mercedes	Campanian - Maastrichtian	?
Mercedes	Campanian - Maastrichtian	?
Mercedes	Campanian - Maastrichtian	?
Guichón	Late Cretaceous	?
Aren Sandstone - Tremp	Late Maastrichtian	Titanosauria?
Tremp	Maastrichtian	Titanosauria?
Aren Sandstone - Tremp	Campanian - Maastrichtian	Titanosauria?
Tremp	Campanian - Maastrichtian	Titanosauria?
Marnes rouges inférieures	Upper Maastrichtian	Titanosauria
Marnes rouges inférieures	Upper Maastrichtian	Titanosauria
Sânpetru	Maastrichitian	Titanosauria
Gaogou	Turonian to Cenomanian	Sauropod?
Xiaguan	Upper Cretaceous	Sauropod?
Zhuyangguan	Upper Cretaceous	Sauropod?
Lameta	Maastrichitian	Titanosauria
Lameta	Maastrichitian	Titanosauria?
Lameta	Maastrichitian	Titanosauria?
Lameta	Maastrichitian	Titanosauria?
Barun-Goyot	Upper Cretaceous	Sauropoda?
?	Aptian?	Lithostrotia
Seonso	Upper Cretaceous	Titanosauria
Sihwa	Aptian	Sauropoda?

Egg and eggshell morphology	Egg spatial distribution	Transport	Sediment and setting	Volcanism	Climate	Vegetation
X	?	AL	-	-	-	-
X	?	AL	-	-	-	-
X	X	AU	X	X	X	X
X	X	AU	X - ?	?	?	?
X	X	AU - PA	X	X	X	X
X	-	AU	X - ?	?	X	?
X	-	AU	X - ?	?	X	?
X	?	AU? - PA?	X	?	?	?
?	?	?	?	?	?	?
?	?	?	X	?	?	?
?	?	?	X	?	?	?
X	?	?	X - ?	-	X	-
X	?		X - ?	-	X	-
X - ?	?	AU? - PA?	X	-	X	?
X - ?	?	AU? - PA?	X	-	X	
X	?	AU? - PA?	X - ?	X	X	?
X	X - ?	AU - PA?	X - ?	X	X	X
X	X - ?	AU? - PA?	X - ?	X	X	?
X	X - ?	AU? - PA?	X - ?	X	X	?
X	X	AU	X	X	X	X
X	X	AU	X	X	X	X
X	X	AU	X	X	X	X
?	?	?	X	-	-	-
?	?	?	X	-	-	-
?	?	?	X	-	-	-
X	X	AU	X	X	X	X
X - ?	X - ?	?	X - ?	X	X	X
X - ?	X - ?	AU - PA?	X - ?	X	X	X
X - ?	X - ?	AU - PA?	X - ?	X	X	X
-	-	-	-	-	-	-
X	-	-	-	-	-	-
X	X	AU - PA?	X	X	X	X
?	X	AU - PA?	X	X	X	-

Reference
Argañaraz et al., 2013
de Valais, Apesteguía, Udrizar Sauthier, 2003; Grellet-Tinner, Fiorelli & Salvador, 2012
Grellet-Tinner & Fiorelli, 2010; Grellet-Tinner, Fiorelli & Salvador, 2012
Carignano, Hechenleitner & Fiorelli, 2013; Hechenleitner et al., 2015
Chiappe et al., 1998; Grellet-Tinner, Chiappe & Coria, 2004; Garrido, 2010; Grellet-Tinner, Fiorelli & Salvador, 2012
Simón, 2006; Salgado et al., 2007; Grellet-Tinner, Fiorelli & Salvador, 2012
Simón, 2006; Salgado et al., 2007; Grellet-Tinner, Fiorelli & Salvador, 2012
Manera de Bianco, 1996; Grellet-Tinner, Fiorelli & Salvador, 2012
Powell, 1993, 1994, 1998; Marquillas, del Papa & Sabino, 2005; Becker et al., 2015
Mourier et al., 1988; Vianey-Liaud et al., 1997
Vianey-Liaud et al., 1997; Sigé et al., 2004
Faccio, Ford & Gancio, 1990; Faccio, 1994; Soto, Pol & Perea, 2011
Faccio, 1994; Soto, Pol & Perea, 2011
Alonso-Zarza, Genise & Verde, 2011; Soto, Pol & Perea, 2011
Soto, Perea & Cambiaso, 2012
Vianey-Liaud & López-Martínez, 1997; López-Martínez, 2000; Sellés & Vila, 2015
López-Martínez, 2000; Sander et al., 2008; Vila, Jackson & Galobart 2010; Vila et al., 2010, 2011; Sellés & Vila, 2015
Sander et al., 1998, 2008; López-Martínez, 2000; Sellés & Vila, 2015
Bravo & Vila, 2005; Vila et al., 2009, 2011; Sellés & Vila, 2015
Cousin & Breton, 2000
Cousin & Breton, 2000
Grellet-Tinner et al., 2012
Zhao, 1994; Liang et al., 2009
Liang et al., 2009
Liang et al., 2009
Wilson et al., 2010
Mohabey, 1998; Fernández & Khosla, 2015
Mohabey, 1998; Fernández & Khosla, 2015
Mohabey, 1996, 1998; Fernández & Khosla, 2015
Zhao & Ding, 1976; Mikhailov, 1991, 1995; Mikhailov, Sabath & Kursanov, 1994
Grellet-Tinner et al., 2011
Huh & Zelenitsky, 2002; Paik, Huh & Kim, 2004; Kim et al., 2009b; Paik, Kim & Huh, 2012
Lee, Yu & Wood, 2001; Lee, 2003; Kim et al., 2009a

References cited in Table S1

Alonso-Zarza AM, Genise JF, Verde M. 2011. Sedimentology, diagenesis and ichnology of Cretaceous and Palaeogene calcretes and palustrine carbonates from Uruguay. *Sedimentary Geology* 236:45–61.

Argañaraz E, Grellet-Tinner G, Fiorelli LE, Krause LM, Rauhut OWH. 2013. Huevos de saurópodos del Aptiano–Albiano, Formación Cerro Barcino (Patagonia, Argentina): Un enigma paleoambiental y paleobiológico. *Ameghiniana* 50:33–50.

Becker TP, Summa LL, Ducea MN, Karner GD. 2015. Temporal growth of the Puna Plateau and its bearing on the post – Salta Rift system subsidence of the Andean foreland basin at 25°30′S. *Geological Society of America Memoir 212*:407–433.

Bravo AM, Vila B. 2005. Dinosaur egg remains from the Upper Cretaceous of Vallcebre Syncline (Berguedà, Barcelona Province). *Revista Española de Paleontología*:49–57.

Carignano AP, Hechenleitner EM, Fiorelli LE. 2013. Hallazgo de ostrácodos (Crustacea) Cretácicos continentales en la Formación Los Llanos, localidad de Tama, La Rioja. *Ameghiniana* 50 (suppl):R39.

Chiappe LM, Coria R, Dingus L, Jackson F, Chinsamy A, Fox M. 1998. Sauropod dinosaur embryos from the Late Cretaceous of Patagonia. *Nature* 396:258–261.

Cousin R, Breton G. 2000. A precise and complete excavation is necessary to demonstrate a dinosaur clutch structure. In: *First International Symposium on dinosaur eggs and babies/Extended abstracts*. 31–42.

Faccio G. 1994. Dinosaurian eggs from the Upper Cretaceous of Uruguay. In: Carpenter K, Hirsch KF, Horner JR eds. *Dinosaur Eggs and Babies*. United States of America: Cambridge University Press,47–55.

Fernández MS, Khosla A. 2015. Parataxonomic review of the Upper Cretaceous dinosaur eggshells belonging to the oofamily Megaloolithidae from India and Argentina. *Historical Biology* 27:158–180.

Garrido AC. 2010. Paleoenvironment of the Auca Mahuevo and Los Barreales sauropod nesting-sites (Late Cretaceous, Neuquén Province, Argentina). *Ameghiniana* 47:99–106.

Grellet-Tinner G, Sim CM, Kim DH, Trimby P, Higa A, An SL, Oh HS, Kim T, Kardjilov N. 2011. Description of the first lithostrotian titanosaur embryo in ovo with Neutron characterization and implications for lithostrotian Aptian migration and dispersion. *Gondwana Research* 20:621–629.

Grellet-Tinner G, Codrea V, Folie A, Higa A, Smith T. 2012. First evidence of reproductive adaptation to “island effect” of a dwarf Cretaceous Romanian titanosaur, with embryonic integument in ovo. *PloS One* 7:e32051.

Grellet-Tinner G, Chiappe LM, Coria RA. 2004. Eggs of titanosaurid sauropods from the Upper Cretaceous of Auca Mahuevo (Argentina). *Canadian Journal of Earth Sciences* 41:949–960.

Grellet-Tinner G, Fiorelli LE. 2010. A new Argentinean nesting site showing neosauropod dinosaur reproduction in a Cretaceous hydrothermal environment. *Nature Communications* 1:32.

Grellet-Tinner G, Fiorelli LE, Salvador RB. 2012. Water vapor conductance of the Lower Cretaceous dinosaurian eggs from Sanagasta, La Rioja, Argentina: Paleobiological and paleoecological implications for South American faveoloolithid and megaloolithid eggs. *Palaios* 27:35–47.

Hechenleitner EM, Fiorelli LE, Grellet-Tinner G, Basilici G, Leuzinger L. 2015. A new titanosaur nesting site from the Los Llanos Formation (Late Cretaceous, La Rioja, Argentina). *Ameghiniana* 52 (suppl):R23.

Huh M, Zelenitsky DK. 2002. Rich dinosaur nesting site from the Createaceous of Bosung County, Chullanam-Do Province, South Korea. *Journal of Vertebrate Paleontology* 22:716–718.

Kim SB, Kim Y, Jo HR, Jeong KS, Chough SK. 2009a. Depositional facies, architecture and environments of the Sihwa Formation (Lower Cretaceous), mid-west Korea with special reference to dinosaur eggs. *Cretaceous Research* 30:100–126.

Kim C-B, Al-Aasm IS, Ghazban F, Chang H-W. 2009b. Stable isotopic composition of dinosaur eggshells and pedogenic carbonates in the Upper Cretaceous Seonso Formation, South Korea: Paleoenvironmental and diagenetic implications. *Cretaceous Research* 30:93–99.

Lee Y-N. 2003. Dinosaur bones and eggs in South Korea. *Memoir of the Fukui Prefectural Dinosaur Museum* 2:113–121.

Lee Y-N, Yu K-M, Wood CB. 2001. A review of vertebrate faunas from the Gyeongsang Supergroup (Cretaceous) in South Korea. *Palaeogeography, Palaeoclimatology, Palaeoecology* 165:357–373.

Liang X, Wen S, Yang D, Zhou S, Wu S. 2009. Dinosaur eggs and dinosaur egg-bearing deposits (Upper Cretaceous) of Henan Province, China: Occurrences, palaeoenvironments, taphonomy and preservation. *Progress in Natural Science* 19:1587–1601.

Lopez-Martinez N. 2000. Eggshell sites from the Cretaceous-Tertiary transition in South-Central Pyrenees (Spain). *First International Symposium on dinosaur eggs and babies/Extended abstracts*.

Manera de Bianco T. 1996. Nueva localidad con nidos y huevos de dinosaurios (Titanosauridae) del Cretácico Superior, Cerro Blanco, Yaminué, Río Negro, Argentina. *Asociación Paleontológica Argentina, Publicación Especial* 4:56–67.

Marquillas R a., del Papa C, Sabino IF. 2005. Sedimentary aspects and paleoenvironmental evolution of a rift basin: Salta Group (Cretaceous-Paleogene), northwestern Argentina. *International Journal of Earth Sciences* 94:94–113.

Mikhailov KE. 1991. Classification of fossil eggshells of amniotic vertebrates. *Acta Palaeontologica Polonica* 36(2):193–230.

Mikhailov KE. 1995. Systematic, faunistic and stratigraphic diversity of Cretaceous eggs in Mongolia: Comparison with China. In: Sun A, Wang Y eds. *Sixth Symposium on Mesozoic Terrestrial Ecosystems and Biota, Short Papers*. Beijing: China Ocean Press, 165–168.

Mikhailov KE, Sabath K, Kurzanov S. 1994. Eggs and nests from the Cretaceous of Mongolia. In: Carpenter K, Hirsch KF, Horner JR eds. *Dinosaur Eggs and Babies*. United States of America: Cambridge University Press,88–115.

Mohabey DM. 1996. Depositional environment of Lameta Formation (Late Cretaceous) of Nand-Dongargaon Inland basin, Maharashtra: the fossil and lithological evidences. *Memoirs-Geological Society of India*: 363-386.

Mohabey DM. 1998. Systematics of indian Upper Cretaceous dinosaur and chelonian eggshells. *Journal of Vertebrate Paleontology* 18:348–362.

Mourier T, Bengtson P, Bonhomme M, Buge E., Cappetta H, Crochet JY, Feist M, Hirsch KF, Jaillard E, Laubacher G, Lefranc JP, Moullade M, Noblet C, Pons D, Rey J, Sige B, Tambareau Y, Taquet P. 1988. The Upper Cretaceous - Lower Tertiary marine to continental transition in the Bagua basin, northern Peru. Paleontology, Biostratigraphy, radiometry, correlations. *Newsletters in Stratigraphy* 19:143–177.

Paik IS, Huh M, Kim HJ. 2004. Dinosaur egg-bearing deposits (Upper Cretaceous) of Boseong, Korea: occurrence, palaeoenvironments, taphonomy, and preservation. *Palaeogeography, Palaeoclimatology, Palaeoecology* 205:155–168.

Paik IS, Kim HJ, Huh M. 2012. Dinosaur egg deposits in the Cretaceous Gyeongsang Supergroup, Korea: Diversity and paleobiological implications. *Journal of Asian Earth Sciences* 56:135–146.

Powell JE. 1993. Primer registro de huevos de dinosaurios del Cretácico Superior del Noroeste Argentino. *1º Jornadas ide Comunicaciones Internas, Fac. de Cs. Nat. e Inst. Miguel Lillo, Universidad Nacional de Tucumán*:56.

Powell JE. 1994. First record of dinosaur eggs in the Upper Cretaceous of northwestern Argentina. *6º Congreso Argentino de Paleontología y Bioestratigrafía, Abstracts*:1.

Powell JE. 1998. Los vertebrados fósiles. In: Gianfrancisco M, Puchulu M, Durango de Cabrera ME, Aceñolaza GF eds. *Geología de Tucumán*. San Miguel de Tucumán: Colegio de Graduados en Ciencias Geológicas de Tucumán, 227–240.

Salgado L, Coria RA, Magalhaes Ribeiro CM, Garrido A, Rogers R, Simón ME, Arcucci AB, Rogers KC, Carabajal AP, Apesteguía S, Fernández M, García RA, Talevi M. 2007. Upper Cretaceous dinosaur nesting sites of Río Negro (Salitral Ojo de Agua and Salinas de Trapalcó-Salitral de Santa Rosa), northern Patagonia, Argentina. *Cretaceous Research* 28:392–404.

Sander PM, Peitz C, Gallemi J, Cousin R. 1998. Dinosaurs nesting on a red beach? *C. R. Acad. Sci. Paris* 327:67–74.

Sander PM, Peitz C, Jackson FD, Chiappe LM. 2008. Upper Cretaceous titanosaur nesting sites and their implications for sauropod dinosaur reproductive biology. *Palaeontographica Abt. A* 284:69–107.

Sellés AG, Vila B. 2015. Re-evaluation of the age of some dinosaur localities from the southern Pyrenees by means of megaloolithid oospecies. *Journal of Iberian Geology* 41:125–139.

Sigé B, Sempere T, Butler RF, Marshall LG, Crochet JY. 2004. Age and stratigraphic reassessment of the fossil-bearing Laguna Umayo red mudstone unit, SE Peru, from regional stratigraphy, fossil record, and paleomagnetism. *Geobios* 37:771–794.

Simón ME. 2006. Cáscaras de huevos de dinosaurios de la Formación Allen (Campaniano-Maastrichtiano), en Salitral Moreno, provincia de Río Negro, Argentina. *Ameghiniana* 43:513–528.

Soto M, Perea D, Cambiaso A. 2012. First sauropod (Dinosauria: Saurischia) remains from the Guichón Formation, Late Cretaceous of Uruguay. *Journal of South American Earth Sciences* 33:68–79.

Soto M, Pol D, Perea D. 2011. A new specimen of *Uruguaysuchus aznarezi* (Crocodyliformes: Notosuchia) from the middle Cretaceous of Uruguay and its phylogenetic relationships. *Zoological Journal of the Linnean Society* 163:S173–S198.

de Valais S, Apesteguía S, Udrizar Sauthier D. 2003. Nuevas evidencias de dinosaurios de la Formación Puerto Yeruá (Cretácico), Provincia de Entre Ríos, Argentina. *Ameghiniana* 40:631–635.

Vianey-Liaud M, Hirsch K, Sahni A, Sigé B. 1997. Late Cretaceous Peruvian eggshells and their relationships with Laurasian and Eastern Gondwanian material. *Geobios* 30: 75-90.

Vianey-Liaud M, Lopez-Martinez N. 1997. Late Cretaceous dinosaur eggshells from the Tremp Basin, southern Pyrenees, Lleida, Spain. *Journal of Paleontology* 71: 1157-1171

Vila B, Galobart À, Oms O, Poza B, Bravo AM. 2009. Assessing the nesting strategies of Late Cretaceous titanosaurs: 3-D clutch geometry from a new megaloolithid eggsite. *Lethaia* 43:197–208.

Vila B, Jackson FD, Fortuny J, Sellés AG, Galobart A. 2010. 3-D modelling of megaloolithid clutches: insights about nest construction and dinosaur behaviour. *PloS one* 5:e10362.

Vila B, Riera V, Bravo AM, Oms O, Vicens E, Estrada R, Galobart À. 2011. The chronology of dinosaur oospecies in south-western Europe: Refinements from the Maastrichtian succession of the eastern Pyrenees. *Cretaceous Research* 32:378–386.

Vila B, Jackson FD, Galobart À. 2010. First data on dinosaur eggs and clutches from Pinyes locality (Upper Cretaceous, Southern Pyrenees). *Ameghiniana* 47:79–87.

Wilson JA, Mohabey DM, Peters SE, Head JJ. 2010. Predation upon hatchling dinosaurs by a new snake from the Late Cretaceous of India. *PLoS Biology* 8:e1000322.

Zhao Z-K. 1994. Dinosaur eggs in China: On the structure and evolution of eggshells. In: Carpenter K, Hirsch KF, Horner JR eds. *Dinosaur Eggs and Babies*. United States of America: Cambridge University Press,184–203.

Zhao Z, Ding S. 1976. Discovery of the dinosaurian egg-shells from Alxa, Ningxia and its stratigraphic significance. *Vertebrata PalAsiatica* 14:42–44.
